# Genome Sequence of *Mycoplasma feriruminatoris* sp. nov., a Fast-Growing *Mycoplasma* Species

**DOI:** 10.1128/genomeA.00216-12

**Published:** 2013-02-21

**Authors:** Anne Fischer, Ivette Santana-Cruz, Michelle Giglio, Suvarna Nadendla, Elliott Drabek, Edy M. Vilei, Joachim Frey, Joerg Jores

**Affiliations:** a International Livestock Research Institute, Nairobi, Kenya; b International Centre for Insect Physiology and Ecology, Nairobi, Kenya; c Institute for Genome Sciences, Baltimore, Maryland, USA; d Institute of Veterinary Bacteriology, University of Bern, Bern, Switzerland

## Abstract

Members of the “*Mycoplasma mycoides* cluster” represent important livestock pathogens worldwide. We report the genome sequence of *Mycoplasma feriruminatoris* sp. nov., the closest relative to the “*Mycoplasma mycoides* cluster” and the fastest-growing *Mycoplasma* species described to date.

## GENOME ANNOUNCEMENT

Species belonging to the “*Mycoplasma mycoides* cluster” represent important livestock pathogens causing substantial economic losses in cattle, goats, and sheep. Recently, a group of *Mycoplasma* strains closely related to the “*M. mycoides* cluster” has been identified by multilocus sequence typing (1). These five strains were isolated from a Rocky Mountain goat and four Alpine ibexes. We assessed the taxonomic position of these strains and suggest that they represent a novel species named *M. feriruminatoris* sp. nov. (J. Jores, M. Heller, C. Schnee, A. Fischer, and J. Frey, unpublished results). This species is the fastest-growing *Mycoplasma* species described so far, with a generation time of less than 30 min. Here we report the genome sequence of *Mycoplasma feriruminatoris* sp. nov. G5847^T^ (=DSM 26019^T^ = NCTC 13622^T^). This genome was sequenced with Illumina GAIIx paired-end sequencing, with reads of about 75 bases. The reads were assembled using Velvet 1.1 (2). The best assembly resulted from a subsample of 140× genome coverage of Illumina reads. The draft genome comprises 92 contigs totaling a length of 1,019,436 bp; 25 contigs that were longer than 10,000 bp covered 89% of the assembly. The *N*_50_ contig size is 46,000 bp.

The average G+C content of the genome is 24.1%. Open reading frames (ORFs) were predicted using Prodigal 2.50 (3). A total of 879 ORFs, 29 tRNAs, and the 23S, 16S, and 5S rRNA operons were predicted. Functional annotation was produced by the Institute for Genome Sciences Annotation Engine (4) (http://ae.igs.umaryland.edu/cgi/index.cgi). The average gene length is 1,058 bp, and the coding density of the genome is 91%. Of the 879 ORFs, 692 have been assigned functions, have received annotations based on membership in a protein family, or have been identified as containing a particular domain. The remaining ORFs are hypothetical. Four genes encode proteins involved in fatty acid and phospholipid metabolism; 29 encode proteins involved in amino acid, purine, pyrimidine, nucleoside and nucleotide, cofactor, prosthetic group, and carrier metabolism; 57 encode proteins involved in central, intermediary, and energy metabolism; and 221 encode proteins involved in DNA metabolism, transcription, protein synthesis, and protein fate. In addition, 123 gene products are involved in transport and binding, 13 have regulatory functions, 10 are involved in signal transduction, 45 are cell envelope lipoproteins, and 63 are involved in cellular processes. Seven genes have mobile element functions. One of them encodes a phage integrase family protein, which has homologs only in *Mycoplasma bovis*, *Mycoplasma agalactiae*, and *Mycoplasma hyorhinis*.

Due to their minimal genome size, mycoplasmas have become model organisms to study fundamental questions of biological life. More recently, they have become the target species in synthetic biology and constructions of synthetic genomes (5, 6). One of the rate-limiting factors in such studies is the slow generation time of most *Mycoplasma* species relative to other microbial organisms. Understanding the basis of the short generation time of *M. feriruminatoris* sp. nov. should accelerate the use of *Mycoplasma* as a template for designer organisms with fully synthetic genomes.

### Nucleotide sequence accession numbers.

This Whole Genome Shotgun project has been deposited at DDBJ/EMBL/GenBank under the accession number ANFU00000000. The version described in this paper is the first version, ANFU01000000.
